# Bulk RNA Sequencing Reveals Signature Differences in Key Cell Signaling Pathways Between Porcine Venous and Arterial Smooth Muscle Cells

**DOI:** 10.3390/ijms262411948

**Published:** 2025-12-11

**Authors:** Kent A. Lee, Wei Li, Unimunkh Uriyanghai, Christine Wai, Huanjuan Su, Anthony Yang, Lianxia Li, Vinay A. Sudarsanam, John S. Poulton, Prabir Roy-Chaudhury, Gang Xi

**Affiliations:** 1 UNC Kidney Center, Division of Nephrology and Hypertension, Department of Medicine, the University of North Carolina at Chapel Hill, Chapel Hill, NC 27599, USA; kentlee@unc.edu (K.A.L.); azyang@email.unc.edu (A.Y.);; 2 BioInfoRx Inc., Madison, WI 53719, USA; weili@bioinforx.com; 3 WG (Bill) Hefner VA Medical Center, Salisbury, NC 28144, USA

**Keywords:** vascular smooth muscle cells (VSMCs), vein, artery, arteriovenous fistulae (AVF), stenosis, dedifferentiation

## Abstract

We recently identified significant differences between porcine arterial and venous smooth muscle cells (ApSMCs and VpSMCs) in the expression of numerous genes and activity of several important signaling pathways. To understand the mechanisms that are responsible for these differences, we performed a genome-wide comparison of VpSMCs and ApSMCs using bulk RNA sequencing. A principal component analysis (PCA) plot and heatmaps revealed a clear separation of the two groups of samples. Using a standard cutoff (≥2-fold change, false discovery rate (FDR) ≤ 0.05), 466 genes were highly expressed in ApSMCs, and 358 genes were highly expressed in VpSMCs. Functional pathway analyses were conducted using the Gene Set Enrichment Analysis (GSEA) tool. The top 15 enriched pathways of the GSEA and Overrepresentation Analysis (ORA) results were detected by comparing the dataset against the Kyoto Encyclopedia of Genes and Genomes (KEGG), Gene Ontology (GO) biological process, GO cellular component, GO molecular function, and WikiPathways databases. Both the GSEA and ORA results revealed that the top enriched pathways are mostly linked to cell cycle, cell structure, and cell differentiation. Further analysis of differentially expressed genes (DEGs) in a specific pathway identified that different sets of genes were utilized to regulate the same pathway between ApSMCs and VpSMCs. For example, in the cell cycle pathway, TGFB1, GADD45A, and TP53 were expressed highly in ApSMCs, while SKP2, PCK1, CDK1, and PPP2CA were expressed highly in VpSMCs. This study identified key differences in the gene expression patterns of two subsets of VSMCs and found that different sets of genes are utilized in specific signaling pathways within the different subtypes of cells, which provides crucial information for developing vein- or artery-specific strategies to prevent corresponding vascular diseases.

## 1. Introduction

Clinic observations have revealed significant differences between arterial vascular smooth muscle cells (VSMCs) and venous VSMCs under different environmental settings, disease conditions, or injuries [1,2]. For example, atherosclerosis mainly occurs in arteries but rarely in veins under normal anatomic conditions [3]. On the other hand, veins have high rates of severe stenosis, vascular occlusive lesions, or increased fibrotic scarring after vascular interventions, such as creation of arteriovenous fistulas (AVFs), balloon angioplasty, or stent placement [2,4,5]. To understand the underlying mechanisms that are responsible for these different phenotypes of arteries and veins, different methodology studies have been conducted, including in vitro cultures of isolated primary venous VSMCs and arterial VSMCs [6,7,8], microarray hybridization [3], and venous and arterial tissue RNA seq analysis (bulk and single cells) [9,10].

Our prior in vitro studies demonstrated different biological functions within these two subtypes of VSMCs using cultured porcine VSMCs, including high proliferation and migration; high susceptibility for dedifferentiation cues, such as platelet-derived growth factor BB (PDGF-BB) treatment; high expression of cell focal adhesive proteins, such as focal adhesion kinase (FAK) and vascular cell adhesion molecule 1 (VCAM-1); low expression of p53, vitronectin, collagen 1A1, and integrin β3 but high expression of fibronectin; high expression of matrix metalloproteinase (MMP)-2, -3, and -9; and strong activation of c-Jun N-terminal kinases (JNKs), P38 mitogen-activated protein kinase (MAPK), and AKT serine/threonine kinase (AKT) in response to PDGF-BB in venous VSMCs, compared to arterial VSMCs [8]. Unlike in vitro cell culture studies, a microarray hybridization approach was employed to reveal the dramatic differences in gene expression patterns and functional response to oxidized low-density lipoprotein (OxLDL) and PDGF-BB between human coronary artery VSMCs and saphenous vein VSMCs. The study demonstrated that venous VSMCs had stronger proliferation/migratory responses to stimuli and also had higher expression of atheroprotective genes at baseline [3]. Using deep single-cell RNA sequencing combined with in situ gene and protein expression analysis in murine heart, aorta, lung, and colon, Muhl L et al. revealed that arterial VSMCs exhibit extensive organotypic heterogeneity, whereas venous VSMCs show similarity across organs [9]. Most recently, using a combination of bulk and single-cell RNA sequencing, proteomic, flow cytometry, and histology, a study demonstrated a 7.8-fold higher proportion of contractile VSMCs in upper arm arteries, as well as a higher abundance of endothelial cells, pericytes, and macrophages, and an increasing trend of fibroblasts in upper arm veins. The study further revealed that activated fibroblasts from veins were the top producers of collagens and highly angiogenic, proinflammatory, and hyper-responders to reactive oxygen species. Most importantly, a higher abundance of fibrillar collagens was found in veins, and more basement extracellular matrix (ECM) proteins were detected in arteries [10].

In most in vitro studies, candidate gene approaches are utilized to examine potential differences between these two subtypes of VSMCs, based on known protein function and their potential significance. We believe a more comprehensive study is necessary to identify the differences in new proteins or new signaling pathways. However, two previous single-cell RNA sequencing studies focused on organotypic heterogeneity and similarity [9] or cell populations with remodeling potential [10]. Therefore, in this study, we pursued a genome-wide comparison of venous porcine VSMCs (VpSMCs) and arterial porcine VSMCs (ApSMCs) using bulk RNA sequencing, mainly focusing on a variety of critical signaling pathways that regulate important cellular biological functions. Importantly, we believe pigs are anatomically and physiologically more like human beings than small rodents.

## 2. Results

### 2.1. Bulk RNA Sequencing Data Quality Assessment

**Figure 1:**
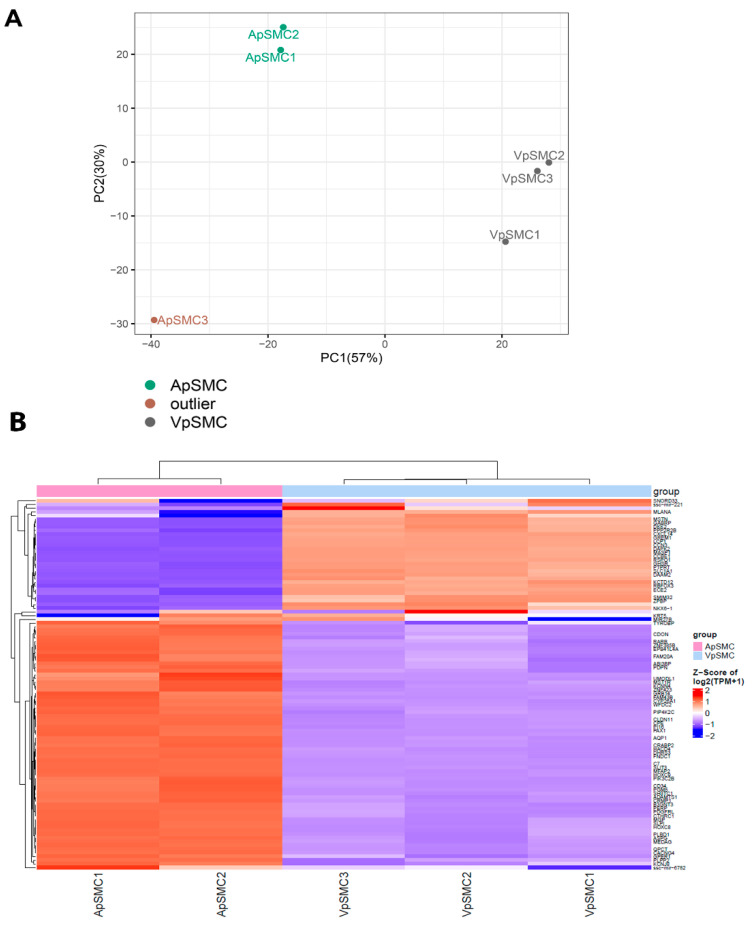
(**A**) Principal component analysis (PCA) of bulk RNA-seq data from arterial (ApSMCs) and venous (VpSMCs) vascular smooth muscle cells (VSMCs). The first two principal components (PC1 and PC2) explain 57% and 30% of the variance, respectively. ApSMCs and VpSMCs form distinct clusters, indicating significant transcriptomic differences. One ApSMCs sample (ApSMC3) is an outlier. (**B**) Heatmap of gene expression data comparing ApSMCs (n = 2) and VpSMCs (n = 3) without the ApSMC3 outlier. Each colored bar represents a different gene, grouped by expression through the dendrogram on the left side. The color of each bar represents the amount of expression measured in transcripts per million (TPM), with red meaning high expression and blue meaning low expression.

To confirm the quality of the RNA-seq dataset for further analysis, exploratory data analysis was performed. A principal component analysis (PCA) demonstrated clear separation between VpSMCs and ApSMCs groups, with the three VpSMCs samples clustering tightly. In contrast, two ApSMCs samples formed a distinct cluster, while ApSMC3 was markedly distant from all other data samples, indicating it is an outlier (Figure 1A). Therefore, two ApSMCs and three VpSMCs samples were included in subsequent downstream analyses.

### 2.2. General Profiles of Differentially Expressed Genes (DEGs) in ApSMCs and VpSMCs

To explore the general profiles of DEGs in ApSMCs and VpSMCs, we first created a heatmap of the top 100 most DEGs across these two subsets of samples (Figure 1B). Columns represent different samples within each group, while rows correspond to individual genes. Hierarchical clustering of samples (top dendrogram) and genes (left dendrogram) was performed based on expression similarity. Gene expression levels are color-coded, with red indicating high expression and blue indicating low expression. The two sample groups showed distinctly different patterns of DEGs, demonstrating the large differences between these two subsets of VSMCs.

**Figure 2:**
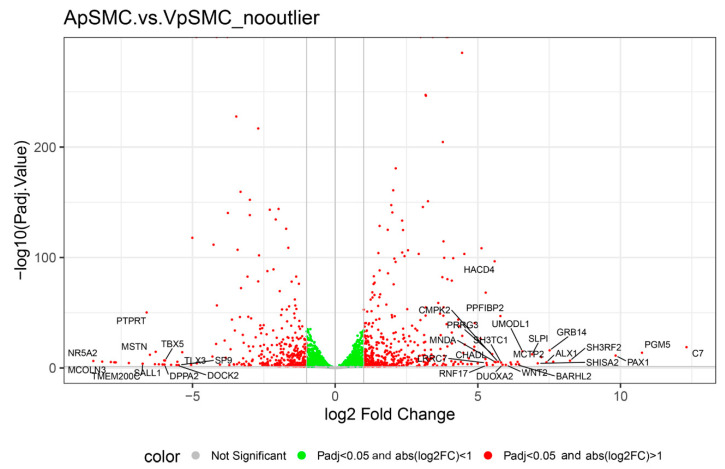
Volcano plot comparing gene expression in ApSMCs and VpSMCs groups without the ApSMC3 outlier. Each dot represents one gene, some of which are labeled based on their importance to this study and biological processes. The *x*-axis shows log2 fold change, the ratio of gene expression between the two groups. Genes below the horizontal grey line represent non-significant genes with a *p*-value < 0.05. The green-colored dots are also not considered due to their low expression values of logFC < 1. The red dots are the most important in this study since they meet the criteria for significance and expression.

To examine the variability in expression data within and among VSMCs types, we generated a volcano plot of DEGs displaying log_2_ fold change (*x*-axis) versus –log_10_(adjusted *p*-value) (*y*-axis) for all genes assessed in the differential expression analysis (Figure 2). Vertical and horizontal dotted lines represent the significance thresholds: |log_2_(fold change)| ≥ 1 and adjusted *p*-value < 0.05. Red points denote DEGs with significantly higher or lower expression in ApSMCs relative to VpSMCs. Genes highlighted with symbols indicate the top DEGs based on absolute log_2_ fold change. The visualization clearly shows that the different sets of genes were expressed highly in these two subsets of VSMCs.

### 2.3. Functional Enrichment Analysis

**Figure 3:**
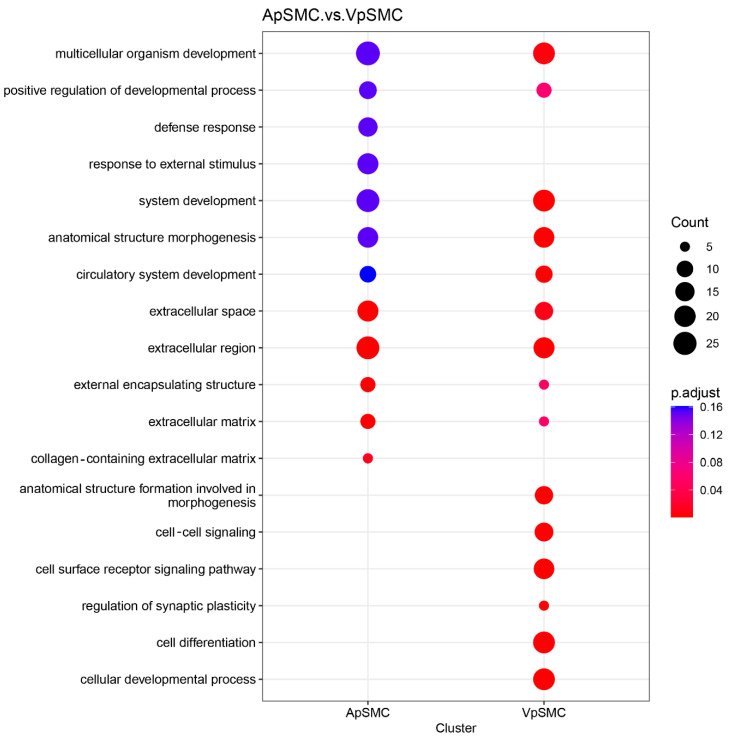
Gene set enrichment analysis (GSEA) dot plot showing the top important processes and a comparison between ApSMCs and VpSMCs groups. The size of the dots represents the gene count, i.e., the number of genes from the process seen in each group. The color of the dots represents the p-adjusted value, with red meaning more significant.

A dot plot of Gene Ontology (GO) terms enriched among the 336 DEGs (log_2_ fold change ≥ 2, FDR < 0.05) was generated using the clusterProfiler R package (version 4.6.2) (Figure 3). The x-axis distinguishes the ApSMCs highly expressed gene list (205 DEGs) and the VpSMCs highly expressed gene list (131 DEGs) clusters, while the y-axis lists enriched GO terms. The color gradient represents adjusted p-values (red: lower, blue: higher), and the dot size reflects the number of annotated DEGs in each category. The results revealed that some categories were highly activated in both subsets of cells, such as multicellular organism development, system development, anatomical structure morphogenesis, and extracellular regions. While some categories were only activated in ApSMCs, such as defense response and response to external stimulus, some were only activated in VpSMCs, such as cell–cell signaling, the cell surface receptor signaling pathway, and cell differentiation. Importantly, different gene expression levels were detected in the same category of enrichment between these two subsets of VSMCs (Table 1), suggesting signature differences in the genes that were utilized.

Functional pathway enrichment was also performed by mapping pig gene identifiers to their corresponding human homologs. Table 2 summarizes the top 15 enriched pathways from the Gene Set Enrichment Analysis (GSEA) using DEGs from the ApSMCs vs. VpSMCs comparison. Table 3 summarizes the top 15 enriched pathways from the Overrepresentation Analysis (ORA) using DEGs from the ApSMCs vs. VpSMCs comparison (log_2_ fold change ≥ 2, FDR < 0.05). The databases queried include Kyoto Encyclopedia of Genes and Genomes (KEGG), Gene Ontology Biological Process, Cellular Component, Molecular Function, and WikiPathways. Both the GSEA and ORA results revealed that the top regulated pathways are primarily involved in the cell cycle, structure, and differentiation.

### 2.4. KEGG Pathway Mapping of DEGs

**Figure 4:**
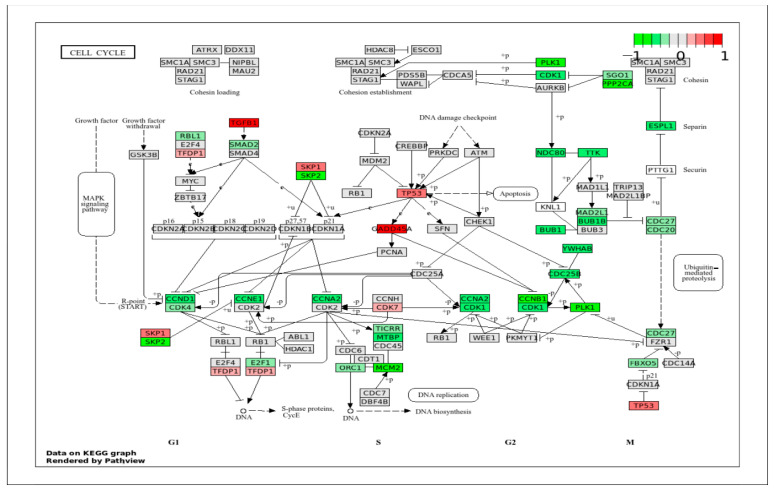
KEGG Pathway of the cell cycle with visualization of gene expression differences between ApSMCs and VpSMCs. Red represents high expression in ApSMCs, while green represents high expression in VpSMCs.

**Figure 5:**
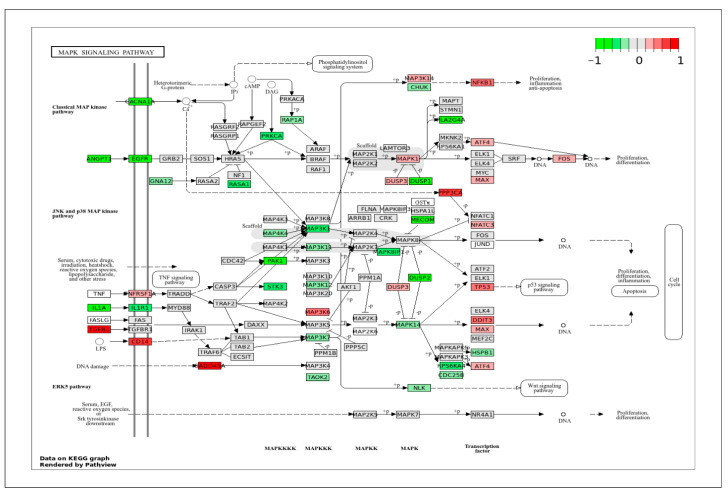
KEGG pathway of the MAPK signaling pathway with visualization of gene expression differences between ApSMCs and VpSMCs. Red represents high expression in ApSMCs, while green represents high expression in VpSMCs.

**Figure 6:**
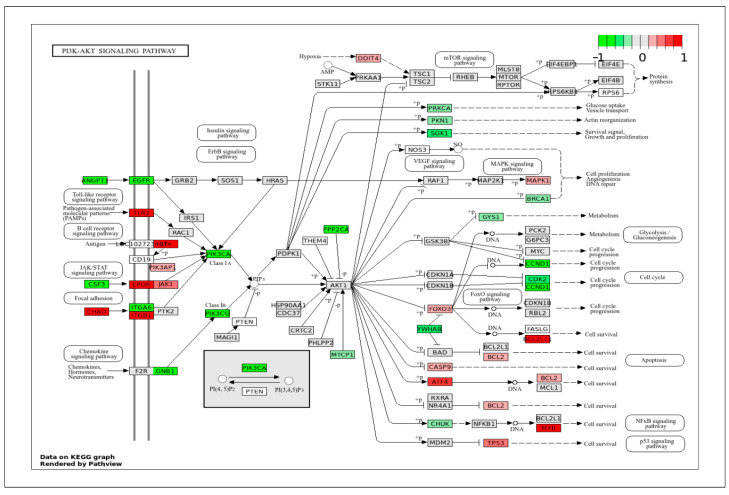
KEGG pathway of the PI3K-AKT signaling pathway with visualization of gene expression differences between ApSMCs and VpSMCs. Red represents high expression in ApSMCs, while green represents high expression in VpSMCs.

**Figure 7:**
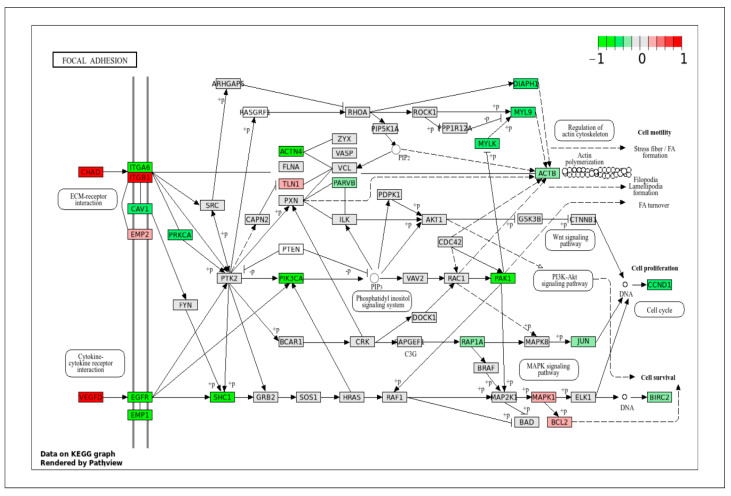
KEGG pathway of focal adhesion with visualization of gene expression differences between ApSMCs and VpSMCs. Red represents high expression in ApSMCs, while green represents high expression in VpSMCs.

**Figure 8:**
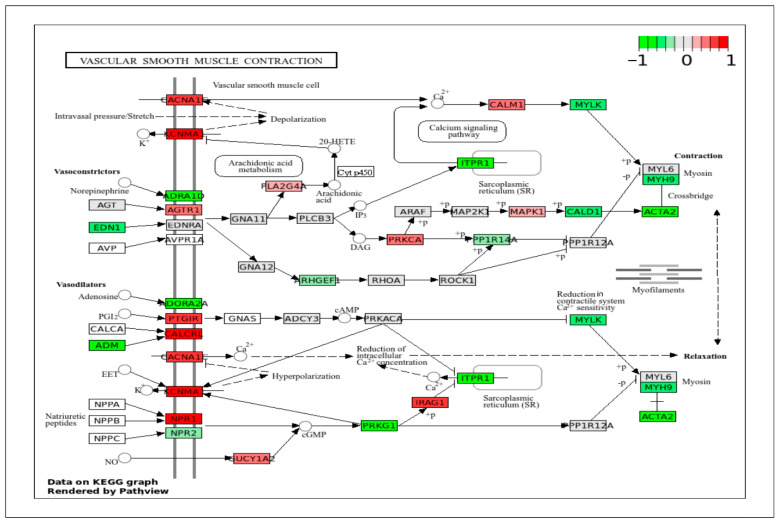
KEGG pathway of vascular smooth muscle contraction with visualization of gene expression differences between ApSMCs and VpSMCs. Red represents high expression in ApSMCs, while green represents high expression in VpSMCs.

**Figure 9:**
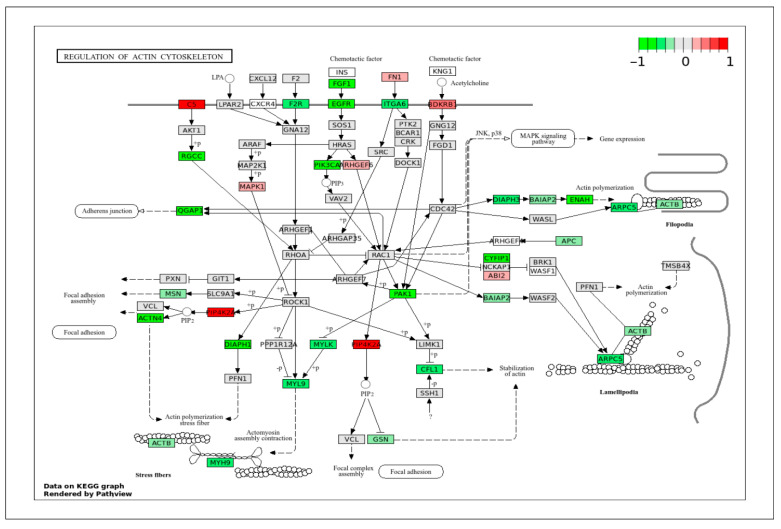
KEGG pathway of actin cytoskeleton regulation with visualization of gene expression differences between ApSMCs and VpSMCs. Red represents high expression in ApSMCs, while green represents high expression in VpSMCs.

**Figure 10:**
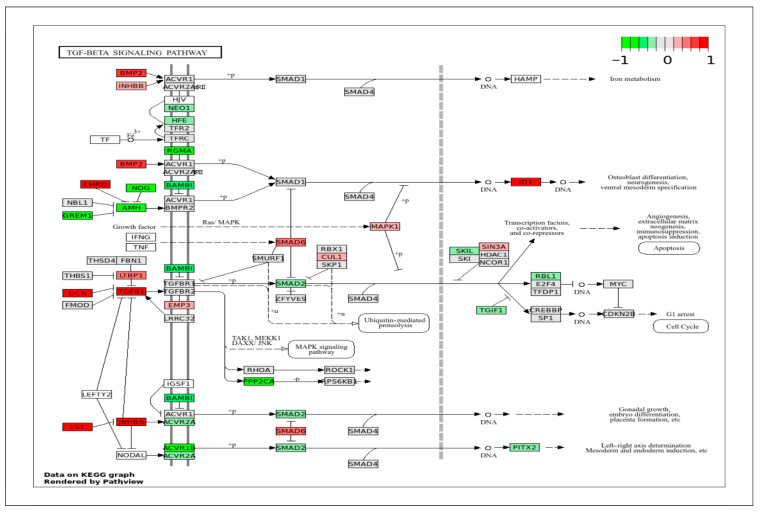
KEGG pathway of TGF-β with visualization of gene expression differences between ApSMCs and VpSMCs. Red represents high expression in ApSMCs, while green represents high expression in VpSMCs.

**Figure 11:**
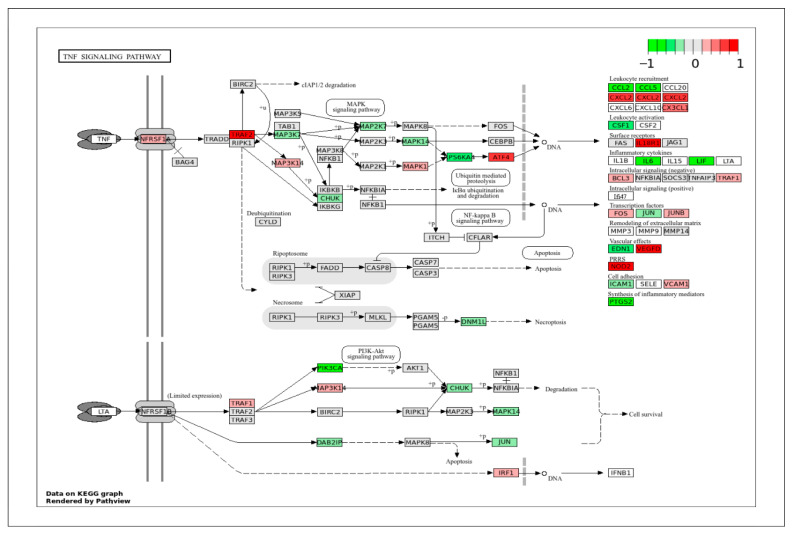
KEGG pathway of the TNF signaling pathway with visualization of gene expression differences between ApSMCs and VpSMCs. Red represents high expression in ApSMCs, while green represents high expression in VpSMCs.

To correlate DEGs with a specific signaling pathway, the DEGs obtained in this study were projected onto the important KEGG pathways that play crucial roles in cell behaviors and biological functions. The pig genes were first converted to their human homologs prior to mapping onto the human pathway schematics provided by the KEGG database (www.genome.jp/kegg/pathway.html (accessed on 28 February 2025)). In all KEGG pathway figures (Figure 4, Figure 5, Figure 6, Figure 7, Figure 8, Figure 9, Figure 10 and Figure 11, Supplemental Figures S1–S4), gene expression is colored by log_2_ fold change with respect to ApSMCs/VpSMCs. Therefore, red indicates genes are highly expressed in ApSMCs, while green indicates genes are highly expressed in VpSMCs.

#### 2.4.1. Signaling Pathways That Mainly Regulate Cell Cycle, Cell Proliferation, and Migration

Our previous study indicated that VpSMCs have higher proliferation and migration ability compared to ApSMCs [8]; therefore, the first categories of signaling pathways examined were related to the cell cycle, cell proliferation, and cell migration. In the cell cycle pathway (Figure 4), the highly expressed genes in ApSMCs included TGFB1, GADD45A, TP53, SKP1, CDK7, and TFDP1. Consistent with our RNA-seq data, we previously found higher levels of p53 protein in ApSMCs. Since p53 plays an important role in the suppression of cell proliferation, low expression of p53 in VpSMCs may contribute to their high proliferation [8]. In VpSMCs, several genes involved in S-phase entry and G2/M transition were highly expressed, including SKP2, PLK1, PPP2CA, CDK1, ESPL1, NDC80, TTK, BUB1B, YMHAB, CDC 25B, CCNB1, PLK1, and MCM2.

In the MAPK signaling pathway (Figure 5), which plays a crucial role in regulating cell proliferation and migration, a set of highly expressed genes in ApSMCs was detected, including TGFβ1, CD14, CADD45A, PPP3CA, and NFKB1. In contrast, a large set of genes was highly expressed in VpSMCs, including IL1A, ANGPT1, EGFR, CACNA1, RASA1, PRKCA, MAP3K1, PAK1, DUSP1, DUSP2, PLA3G4A, MECOM, etc. These genes belong to MAPKKKK, MAPKKK, MAPKK, MAPK, or related transcription factors.

In the PI3K-AKT signaling pathway (Figure 6), which also modulates cell proliferation, the cell cycle, and migration, we identified several highly expressed genes in ApSMCs, such as CHAD, TLR2, EPOR, ITGB1, ATF4, BCL2L11, and MYB. The set of genes highly expressed in VpSMCs included ANGPT1, CSF3, EGFR, ITGA6, GNB1, PIK3CA, PIK3CG, PPP2CA, SGK1, YWHAB, CCND1, and CDK2.

#### 2.4.2. Signaling Pathways That Regulate ECM Organization and Vascular Contractile Function

A major category that contains many DEGs and important pathways is the ECM, the cytoskeleton, and their respective functions. Structural integrity and contractile function are the important features of VSMCs. Differences in cytoskeletal dynamics and ECM interactions help to explain the mechanical demands of arterial versus venous environments. The focal adhesion pathway (Figure 7) showed increased expression of genes that were involved in binding and cell cycle signaling, such as ITGA6, CAV1, EGFR, EMP1, SHC1, ACTN4, PIK3CA, DIAPH1, MYLK, PAK1, and CCND1 in VpSMCs. Some of these genes are also involved in the PI3K-AKT signaling pathway, as mentioned above. In contrast, in ApSMCs, the highly expressed genes included CHAD, VEGFD, and ITGB1, which are more involved in ECM regulation and deposition.

DEGs projected in the ECM–receptor interaction pathway (Supplemental Figure S1) revealed that the highly expressed genes in ApSMCs were COL1A1, LAMA3, COMP, FERM1, IBSP, and ITGB4, while SPP1, ITGA3, ITGA6, ITGA7, ITGA9, TNC, SPP1, and SDC1 were expressed highly in VpSMCs. Most of these genes play important roles in maintaining cell structural integrity and vascular contractile functions.

DEGs data projected in the vascular smooth muscle contraction pathway (Figure 8) showed high expression of contractile elements, such as CACNA1, KCNMA1, CALCRL, and NPR1, in ApSMCs. The highly expressed genes in VpSMCs were EDN1, ADM, ADRA1D, ADORA2A, PRKG1, ITPR1, MYLK, MYH9, CALD1, and ACTA2. Many of these genes are involved in blood pressure regulation and the vasoconstrictor and vasodilator pathways, but they were very different between the two subsets of cells.

The actin cytoskeleton plays a crucial role in regulating the contractility of VSMCs. DEGs data projected in the regulation of the actin cytoskeleton pathway (Figure 9) showed a high expression of C5 and PIP4K2A genes in ApSMCs, whereas highly expressed FGF1, EGFR, ITGA6, RGCC, PIK3CA, ACTN4, DIAPH1, PAK1, ENAH, CYF1P1, and ARPC5 were detected in VpSMCs. Most genes coordinate filament assembly, stress fiber formation, and contractile function.

#### 2.4.3. Signaling Pathways That Mainly Regulate VSMCs Inflammation and Cytokine Secretion

Another important category that contains many DEGs is immune response and stress signaling. VSMCs contribute to immune signaling during injury, remodeling, and disease. Understanding how these responses differ across vessel types can explain their role in many inflammation-driven conditions. In the TGF-β signaling pathway (Figure 10), ApSMCs highly expressed DCN, FST, CHRD, BMP2, TGFB1, INHBA, and ID1, while VpSMCs highly expressed GREM1, NOG, AMH, RGMA, BAMB1, ACVR1B, and PPP2CA. Interestingly, many of the signal transducers, such as the SMAD genes, were expressed at normal levels in both subtypes of cells. Another important pathway that involves an immune response is the TNF signaling pathway (Figure 11). The DEGs-projected results showed an increased expression of TRAF2, ATF4, CXCL2, IL1BR1, VEGFD, and NOD2 in ApSMCs. These genes mediate downstream inflammatory responses, apoptosis, and NF-κB pathway modulation. Meanwhile, PIK3CA, PS6KA, CCL2, CCL5, CSF1, IL6, LIF, and PTGS2 were expressed highly in VpSMCs. In addition to mediating cellular immune responses, both pathways have been shown to regulate VSMCs phenotypic switch and vascular remodeling.

In addition, the Toll-like receptor (TLR) signaling pathway also plays a crucial role in mediating the immune response in VSMCs. The DEGs-projected results showed that TLR1, TLR2, TLR4, and TLR6 were expressed highly in ApSMCs, while PIK3CA, CTSK, MAPK14, SPP1, IL6, and CCL5 were expressed highly in VpSMCs (Supplemental Figure S2). The TLR signaling pathway not only regulates cellular immune response, leading to the production of inflammatory cytokines (e.g., IL-6) [11], but also involves downstream pathways, such as MAPK and PI3K/AKT [12].

#### 2.4.4. Signaling Pathways That Mainly Regulate VSMCs Phenotypic Switch and Vascular Remodeling

Regarding VSMCs phenotypic switch modulation, the Wnt signaling pathway (Supplemental Figure S3) is an important pathway that is able to promote VSMCs proliferation and migration and VSMCs differentiation into osteogenic cells, leading to vascular calcification [13,14,15]. The DEGs-projected data showed that WIF1, WNT2, SERPINF1, FZD2, ROR1, CCDC88CC, REBBP, TCF7, and NFATC1 were highly expressed in ApSMCs, while NOTUM, RSPO3, FRZB, DKK2, WNT5A, BAMBI, GPC4, NKD1, SENP2, DAAM1, FOSL1, CCND1, and CCN4 were expressed highly in VpSMCs.

Regarding vascular remodeling, the cellular senescence pathway (Supplemental Figure S4) is one of the pathways that also needs to be included because senescent VSMCs contribute to overall vascular remodeling, stiffening, and vascular diseases [16]. The DEGs projected in this pathway revealed that TGFB1, TRPV4, PPP3CA, NFATC1, and GADD45A were expressed highly in ApSMCs, while in VpSMCs, PIK3CA, IL1A, CDK4, CCND1, CCNE1, CCNB1, IL6, and SERPINE1 were expressed highly.

## 3. Discussion

Growing evidence suggests that porcine species are excellent models for the study of human pathophysiology for multiple reasons. First, pigs are anatomically and physiologically more like human beings than small rodents, such as mice and rats [17,18]. Second, pigs are genetically three-fold closer to humans than mice [19]. Third, although primates are more similar to humans than pigs, there are increasing societal concerns about primate models. Fourth, pigs and humans have similar drug metabolism profiles, which makes pigs a better model than other animals for human drug efficacy and toxicity studies [20]. Lastly, the similarity between pigs and humans has been demonstrated by a recent engineered porcine kidney xenotransplantation study (https://www.nytimes.com/2025/02/07/health/fourth-pig-kidney-transplant.html# (accessed on 24 November 2025)). One recipient lived with a porcine kidney for 9 months. Although it was removed due to declining function, the patient is still alive. Therefore, we believe pigs are a good biomedical research model.

Using bulk RNA sequencing, we investigated the genome-wide differences in the gene expression of primary porcine arterial and venous VSMCs. Based on a standard cutoff (≥2-fold change, FDR ≤ 0.05), 466 genes were highly expressed in ApSMCs, and 358 genes were highly expressed in VpSMCs. Functional pathway analyses were conducted using the Gene Set Enrichment tool. Both the GSEA and ORA results revealed that the top regulated pathways were mostly involved in the cell cycle, cell structure, and cell differentiation. In addition, further analysis of DEGs in specific pathways, we identified that different sets of genes were utilized in the same pathway between ApSMCs and VpSMCs.

Consistent with our previous in vitro study [8], the current bulk RNA sequencing results also demonstrated a major difference between ApSMCs and VpSMCs in pathways regulating cell cycle progression and cell proliferation. VpSMCs showed broad high expression of genes associated with DNA replication, mitosis, and cell cycle checkpoints, such as SKP2, CDK1, CDC25B, and CCNB1, suggesting an increase in cell proliferation and potential vascular remodeling. In contrast, ApSMCs demonstrated increased expression of pathways related to growth arrest, DNA damage response, and cell cycle inhibition, such as TGFβ1, GADD45A, and TP53. These data support our previous observation that VpSMCs proliferate faster than ApSMCs. Similarly, in the MAPK and PI-3/AKT signaling pathways, different gene sets were identified as mediators of these signaling pathways between the two subtypes of cells, suggesting that both cells activate MAPK kinase and PI3K/AKT via multiple mechanisms.

We previously observed that VpSMCs formed stronger focal adhesions and had higher migration ability [8]. This study revealed that many more focal adhesion proteins were expressed highly in VpSMCs, such as ITGA6, CAV1, EMP1, ACTN4, SHC1, MYLK, and DIAPH1, which regulate integrin signaling, FAK activity, the actin cytoskeleton, and polymerization, promoting cell migration and linking integrin signaling to the MAPK and PI3K pathways. When the DEGs data were projected onto the ECM–receptor interaction, a similar amount but a different kind of gene expression was revealed between the two subtypes of cells. For example, COL1A1, LAMA3, FERMT1, and ITGB4 were expressed highly in ApSMCs. Our previous study confirmed that COL1A1 was expressed highly in ApSMCs [8]. In VpSMCs, SPP1, ITGA3, ITGB6, ITGB7, ITGB9, and SDC1 were expressed highly, indicating that different sets of genes are utilized to mediate the same cellular functions or different functions. Importantly, a recent study revealed that increased osteopontin (SPP1) was able to drive vascular cell activation in failed AVF [21].

Previous studies have shown that cytokines play crucial roles in mediating VSMCs behaviors and functions [22,23]. In this study, we focused on the TGFβ, TNFα, and Toll-like receptor signaling pathways. To negatively regulate the TGFβ signaling pathway, different sets of genes were utilized. For example, DCN, FST, and CHRD were expressed highly in ApSMCs, whereas GREM1, NOG, BAMBI, and PPP2CA were expressed highly in VpSMCs. In addition, BMP2 was expressed highly in ApSMCs, which plays a key role in mediating bone and cartilage formation and may contribute to severe calcification in arteries under certain disease conditions, such as uremic conditions. Indeed, our previous study observed a high expression of early osteogenesis markers, such as Runx2 and ALP, in ApSMCs treated with uremic pig serum [24]. Interestingly, to regulate the activity of a TGFβ family member, Activin, ACVR1B was highly expressed in VpSMCs, while INHBA was highly expressed in ApSMCs, which is another good example of the utilization of different genes to mediate the same function in these two subtypes of cells. Importantly, ACVR1B has been shown to mediate alternative non-canonical intracellular signaling pathways, including the P38 MAPK, extracellular signal-regulated kinases 1/2 (ERK1/2), and JNKs, to regulate cell migration and differentiation [25]. Of note, we previously found that activated P38 MAPK, ERK1/2, and JNKs in VpSMCs mediate PDGF-BB signaling [8].

One of our prior studies revealed that VpSMCs are susceptible to PDGF-BB-induced cell dedifferentiation [8], which may be favorable for vascular remodeling or developing vascular diseases, such as neointimal hyperplasia and stenosis in the venous segment in AVF. On the other hand, our prior study also demonstrated that ApSMCs were susceptible to uremic serum-induced expression of early osteogenesis markers, such as Runx2 and ALP [24]. To understand the underlying mechanisms, we focused on the Wnt signaling pathway and the cellular senescence pathway. A previous study suggested the important role of the Wnt signaling pathway in vascular calcification [26]. The study revealed that multiple Wnt positively regulating genes, such as WNT2, FZD2, ROR1, CCDC88CC, REBBP, TCF7, and NFATC1, but only a couple of negatively regulating genes, such as WIF1 and SERPINF1, were highly expressed in ApSMCs. Meanwhile, several Wnt negative regulators, such as RSPO3, FRZB, DKK2, and NKD1, were expressed highly in VpSMCs. In contrast, the Wnt pathway positive regulators that were highly expressed in VpSMCs were mainly related to the modulation of cell proliferation and differentiation, such as FOSL1, CCND1, and CCN4. Furthermore, VSMCs senescence has been shown to be highly associated with vascular remodeling and a variety of vascular diseases, such as atherosclerosis and hypertension [27]. Interestingly, our study revealed that not only TGFβ but also several calcium or calcium/calmodulin-dependent phosphatase-regulated genes, such as TRPV4, PPP3CA, and NFATC1, were highly expressed in ApSMCs, suggesting the importance of this pathway in mediating vascular calcification and atherosclerosis, which have been shown to mainly occur in arteries [28]. In contrast, although several cellular senescence promoters, such as IL1A, IL6, and SERPINE1, were expressed highly in VpSMCs, several negative regulators, such as CDK4, CCND1, CCNE1, and CCNB1, were also expressed highly, which may balance the development of cellular senescence in venous VSMCs. However, all these findings need to be confirmed via in vivo studies or clinical studies in the future.

Regarding the implications of the DEGs that were detected in this study, we believe many of them are associated with several vascular diseases, such as deep venous thrombosis (DVT) and peripheral arterial disease (PAD). Specifically, we identified DEGs involved in enhanced VSMCs proliferation, migration, and dedifferentiation, which contribute to the stenosis that occurs in veins. As we know, severe narrowing of vessels is a major contributor to thrombosis; these changes in stenosis-promoting processes may contribute to DVT. In addition, enhanced inflammatory signaling is another important factor for thrombosis. This study demonstrated that Il-6, LIF, CCL2, and CCL5 were highly expressed in venous VSMCs. Furthermore, HBEGF was highly expressed in venous VSMCs. HBEGF can function through EGFR to serve as a chemoattractant for cells that express tissue factors, which, in turn, can promote a pro-coagulant state and contribute to DVT formation. Meanwhile, some DEGs may contribute to PAD, such as high expression of BMP2 and genes that positively regulate Wnt signaling in ApSMCs. Furthermore, VSMCs senescence is highly associated with vascular remodeling and a variety of vascular diseases, such as atherosclerosis and hypertension. This study revealed that not only TGFβ but also several calcium or calcium/calmodulin-dependent phosphatase-regulated genes, such as TRPV4, PPP3CA, and NFATC1, were highly expressed in ApSMCs, suggesting the importance of this pathway in mediating vascular calcification and atherosclerosis, which has been shown to mainly occur in arteries.

In summary, this study revealed hundreds of DEGs in both venous and arterial VSMCs via bulk RNA sequencing analysis. Functional pathway analyses were conducted using the Gene Set Enrichment tool. The top 15 enriched pathways in the GSEA and ORA results were detected using a dataset comparison, which were mainly involved in biological processes like the cell cycle, cell structure formation, and cell differentiation. Most importantly, in all the examined signaling pathways, different gene sets were identified as mediators of similar cell biological behaviors and functions between these two subtypes of VSMCs, which is crucial information for developing vein- or artery-specific strategies to treat corresponding diseases. For instance, genes related to the cell cycle and cell differentiation pathways, such as SKP2, CDK1, and ACVR1B, can be targeted in veins to prevent intimal hyperplasia after injury in venous segments of AVF or AVG settings. In addition, genes related to the TGFβ pathway, such as BMP2 and TRPV4, the Wnt pathway, and the cell senescence signaling pathway can be targeted in arteries to prevent vascular calcification or atherosclerosis under diabetic or uremic conditions.

## 4. Methods

### 4.1. Cell Culture and RNA Isolation

The animal study protocol was approved by the University of North Carolina at Chapel Hill IACUC (Chapel Hill, NC, USA) (IACUC ID: 22069 on 28 April 2022). The pig was housed at the UNC–Chapel Hill animal facility, which aligns with IACUC and USDA regulations that prioritize social interaction and animal welfare. ApSMCs and VpSMCs were isolated from the carotid artery and jugular vein of a 4-month-old female Yorkshire pig (62 kg), respectively. The cells were cultured with growth medium (DMEM containing 5 mM glucose and 10% FBS plus 1% penicillin/streptomycin). All experiments were performed on both cells with passage 6. When cells reached 100% confluency, total RNA from 3 plates of each type of cell was isolated using an RNeasy plus mini kit (Qiagen, Germantown, MD, USA) for bulk RNA sequencing (AZENTA life science, Burlington, MA, USA).

### 4.2. RNA-Seq Data Analysis Methods

For the bioinformatics analysis, sequencing reads were aligned to the *Sus scrofa* reference genome (Sscrofa11.1 assembly) using the Subjunc aligner from the Subread package (version 2.0.3) [29]. Gene-level quantification was performed using the featureCounts function within the same package [29]. The sequencing quality of individual samples was assessed using FASTQC (version 0.12.0) [30]. Differential gene expression analysis was conducted using the Bulk RNA View pipeline implemented in the BxGenomics platform, which leverages DESeq2 (version 1.34.0) [31,32]. Whether all datasets were included in the downstream analysis depended on RNA sequencing data quality assessments.

### 4.3. Functional Enrichment Analysis

#### 4.3.1. GO Term Enrichment Analysis

GO enrichment analysis was conducted using the R package clusterProfiler (v4.6.2) [33]. Differentially expressed genes were tested against the GO database (org.Ss.eg.db, v3.18.0) using a hypergeometric test. *p*-values were adjusted for multiple comparisons using the Benjamini–Hochberg method [34], and GO terms with an adjusted *p*-value (FDR) < 0.05 were considered significantly enriched.

#### 4.3.2. Gene Set Enrichment Analysis (GSEA)

GSEA was performed using the fgsea R package (v1.24.0) and the Bulk RNA view tool from the BxGenomics platform to identify significantly enriched gene sets among ranked genes. Genes were pre-ranked by log_2_ fold change, and enrichment scores were calculated using a fast implementation of the GSEA algorithm [35]. Adjusted *p*-values (FDR) < 0.25 were considered significant. Gene sets were obtained by mapping the human gene sets to pig orthologs using NCBI and Ensembl IDs.

#### 4.3.3. Overrepresentation Analysis (ORA)

ORA was performed using the Bulk RNA view tool from the BxGenomics platform to identify biological pathways significantly enriched among differentially expressed genes. Gene sets were tested using a hypergeometric test implemented in R (v4.1.3), *p*-values were adjusted for multiple testing using the Benjamini–Hochberg method.

#### 4.3.4. KEGG Pathway Mapping of DEGs Between ApSMCs and VpSMCs

DEG fold changes were mapped onto the KEGG pathway diagram [36] using the R package Pathview (v1.38.0) with the Bulk RNA view tool from the BxGenomics platform.

## Figures and Tables

**Table 1:** Different genes were involved in the same GO term enrichment categories in ApSMCs and VpSMCs.

Categories	Cell Subtypes	Involved Genes
Multicellular organism development	ApSMCs	TYROBP, PDPN, MFAP2, NTRK3, ANGPT4, CPE, S1PR3, SYK, HMX3, CHL1, CLDN11, VEGFD, PDLIM3, WNT2, LRRN3, EPHA3, GPER1, AQP1, IGF2, CDH5, ISLR2, ALX1, MGP, SPINK2, HOXC5, IFN-OMEGA-3/4
	VpSMCs	GSC, SALL1, CCBE1, SLC1A1, CCN3, ANGPT1, DOK5, TBX5, GPM6B, WT1, DLX1, PTN, BMPER, GHSR, LZTS1, DPPA2, WNT5A, CBLN1, CLDN5, FGF5, PLN
Anatomical structure morphogenesis	ApSMCs	TYROBP, PDPN, MFAP2, ANGPT4, CPE, SYK, HMX3, CHL1, VEGFD, ADAMTS15, WNT2, EPHA3, AQP1, IGF2, CDH5, ISLR2, ALX1, SPINK2
	VpSMCs	GSC, SALL1, CCBE1, SLC1A1, CCN3, ANGPT1, TBX5, WT1, ZPBP, DLX1, PTN, BMPER, TNNT1, LZTS1, WNT5A, CBLN1, CLDN5, FGF5
Circulatory system development	ApSMCs	NTRK3, ANGPT4, CPE, SYK, VEGFD, PDLIM3, WNT2, GPER1, AQP1, CDH5
	VpSMCs	SALL1, CCBE1, SLC1A1, CCN3, ANGPT1, TBX5, WT1, BMPER, WNT5A, CLDN5, PLN
Extracellular space	ApSMCs	DCN, SERPINB10, ORM1, ANGPT4, CPE, CP, VEGFD, ADAMTS15, WNT2, LRRN3, C7, TIMP3, WFDC2, IGF2, SLPI, IFN-OMEGA-3/4, LOC100157354, PLBD1
	VpSMCs	C1QL4, CCBE1, ANGPT1, CPXM1, SERPINI1, MASP1, MSTN, PTN, BMPER, TNFSF10, DKK2, WNT5A, FGF5
Extracellular region	ApSMCs	DCN, MFAP2, SERPINB10, ORM1, ANGPT4, CPE, CP, ADAMTS1, VEGFD, MDK, ADAMTS15, WNT2, LRRN3, C7, WIF1, TIMP3, WFDC2, IGF2, MGP, SLPI, IFN-OMEGA-3/4, LOC100157354, PLBD1
	VpSMCs	C1QL4, CCBE1, CCN3, ANGPT1, CPXM1, SDC1, SERPINI1, MASP1, MMP1, PON1, ZPBP, MSTN, PTN, BMPER, TNFSF10, DKK2, WNT5A, CBLN1, FGF5

**Table 2:** Top 15 enriched pathways identified with GSEA analysis.

Rank	Gene Set	P.Val	P.Adj	NES	Size
1	GOMF_EXTRACELLULAR_MATRIX_STRUCTURAL_CONSTITUENT	2.71 × 10^−7^	0.00148	2.09	109
2	GOBP_REGULATION_OF_NUCLEAR_DIVISION	0.00000809	0.0184	−2	114
3	GOCC_COLLAGEN_CONTAINING_EXTRACELLULAR_MATRIX	0.0000267	0.0292	1.74	281
4	GOMF_LAMININ_BINDING	0.0000855	0.0586	−1.99	25
5	GOBP_SKELETAL_SYSTEM_DEVELOPMENT	0.000123	0.0746	1.62	392
6	GOCC_SCHAFFER_COLLATERAL_CA1_SYNAPSE	0.000170	0.0931	−1.91	66
7	GOBP_NEGATIVE_REGULATION_OF_GLIAL_CELL_DIFFERENTIATION	0.000219	0.109	−1.96	19
8	GOBP_LOCOMOTION	0.000269	0.123	−1.36	834
9	GOBP_EPITHELIUM_DEVELOPMENT	0.000346	0.146	−1.33	829
10	GOBP_POSTSYNAPTIC_MODULATION_OF_CHEMICAL_SYNAPTIC_TRANSMISSION	0.000458	0.152	−1.94	16
11	GOBP_POSITIVE_REGULATION_OF_NUCLEAR_DIVISION	0.000422	0.152	−1.87	38
12	GOBP_G_PROTEIN_COUPLED_RECEPTOR_SIGNALING_PATHWAY	0.000443	0.152	−1.51	380
13	GOBP_MITOTIC_SISTER_CHROMATID_SEGREGATION	0.000666	0.174	−1.66	167
14	GOBP_BLOOD_VESSEL_MORPHOGENESIS	0.000646	0.174	−1.48	468
15	WP_GDNF_RET_SIGNALING_AXIS	0.00106	0.193	−1.89	19

**Table 3:** Top 15 enriched pathways (fold change ≥4, FDR < 0.05, both directions) identified with ORA analysis.

Rank	Gene Set	P.Val	P.Adj	Fold Enrich	SetNum All	DE Direction
1	GOCC_EXTERNAL_ENCAPSULATING_STRUCTURE	1.81 × 10^−15^	2.1 × 10^−11^	4.76	564	Both
2	GOCC_COLLAGEN_CONTAINING_EXTRACELLULAR_MATRIX	2.27 × 10^−14^	1.31 × 10^−10^	5.11	430	Both
3	GOBP_ANIMAL_ORGAN_MORPHOGENESIS	1.92 × 10^−10^	7.43 × 10^−7^	2.82	1042	Both
4	GOBP_ANATOMICAL_STRUCTURE_FORMATION_INVOLVED_IN_MORPHOGENESIS	1.1 × 10^−8^	0.0000320	2.45	1259	Both
5	GOBP_CIRCULATORY_SYSTEM_DEVELOPMENT	1.48 × 10^−8^	0.0000343	2.43	1224	Both
6	GOBP_CELL_MOTILITY	2.27 × 10^−8^	0.0000439	2.15	1813	Both
7	GOBP_REGULATION_OF_MULTICELLULAR_ORGANISMAL_DEVELOPMENT	3.6 × 10^−8^	0.0000596	2.25	1484	Both
8	GOBP_VASCULATURE_DEVELOPMENT	4.79 × 10^−8^	0.0000622	2.75	832	Both
9	GOMF_SIGNALING_RECEPTOR_BINDING	4.83 × 10^−8^	0.0000622	2.36	1495	Both
10	GOBP_CELL_CELL_SIGNALING	5.75 × 10^−8^	0.0000667	2.17	1713	Both
11	GOBP_NEGATIVE_REGULATION_OF_MULTICELLULAR_ORGANISMAL_PROCESS	6.45 × 10^−8^	0.0000680	2.47	1249	Both
12	GOBP_OLFACTORY_BULB_INTERNEURON_DIFFERENTIATION	1.07 × 10^−7^	0.000103	32.06	13	Both
13	GOBP_ENZYME_LINKED_RECEPTOR_PROTEIN_SIGNALING_PATHWAY	2.74 × 10^−7^	0.000245	2.4	1022	Both
14	GOCC_CELL_SURFACE	3.65 × 10^−7^	0.000302	2.85	903	Both
15	GOBP_REGULATION_OF_SYSTEM_PROCESS	5.99 × 10^−7^	0.000453	3.17	561	Both

## Data Availability

The authors declare that all data supporting the findings of this study will be available from the corresponding authors upon request.

## Supplementary Materials

The following supporting information can be downloaded at: https://www.mdpi.com/article/10.3390/ijms262411948/s1.
