# Supplementary material for: Bulk RNA Sequencing Reveals Signature Differences in Key Cell Signaling Pathways Between Porcine Venous and Arterial Smooth Muscle Cells
Source: Int J Mol Sci. 2025 Dec 11;26(24):11948. doi: 10.3390/ijms262411948 (PMC12732443; doi:10.3390/ijms262411948)
Supplement: Supplementary file 1 [file ijms-26-11948-s001.zip › ijms-3944319-supplementary.pdf]

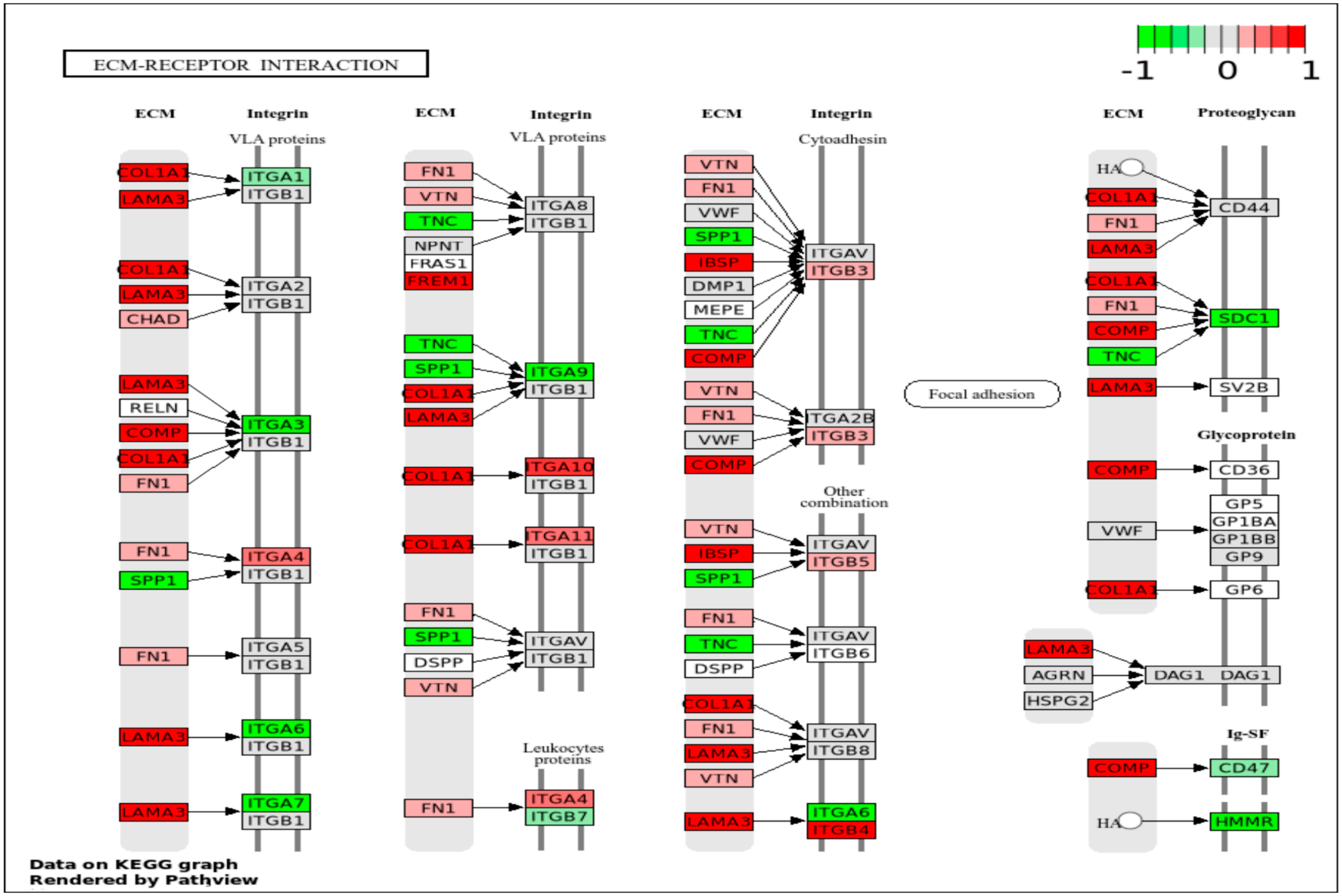

Supplemental Figure S1: KEGG Pathway of the ECM-receptor interaction with visualization of gene expression differences between ApSMCs and VpSMCs. Red represents high expression in ApSMCs while green represents high expression in VpSMCs.

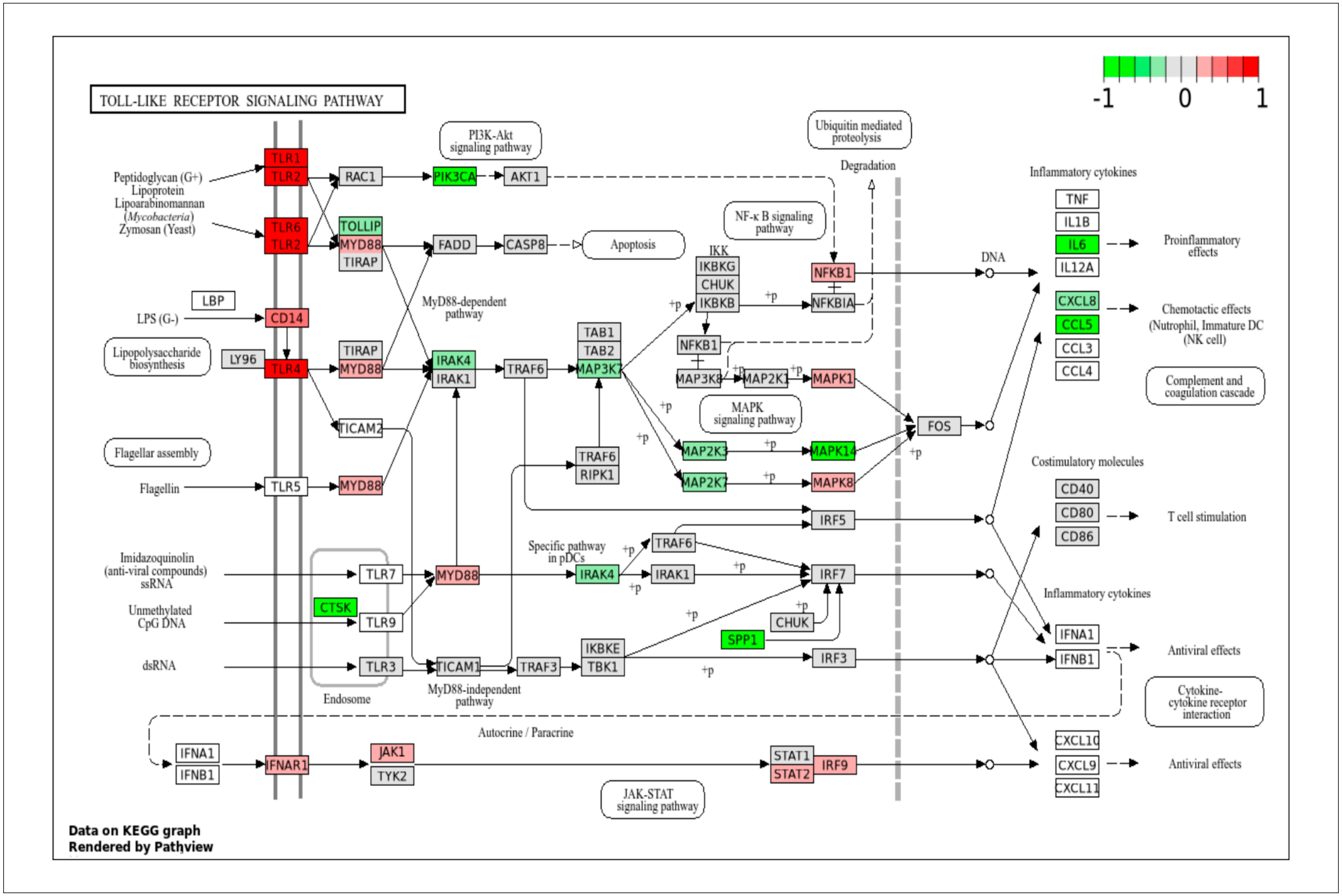

Supplemental Figure S2: KEGG Pathway of the Toll-like receptor signal pathway with visualization of gene expression differences between ApSMCs and VpSMCs. Red represents high expression in ApSMCs while green represents high expression in VpSMCs.

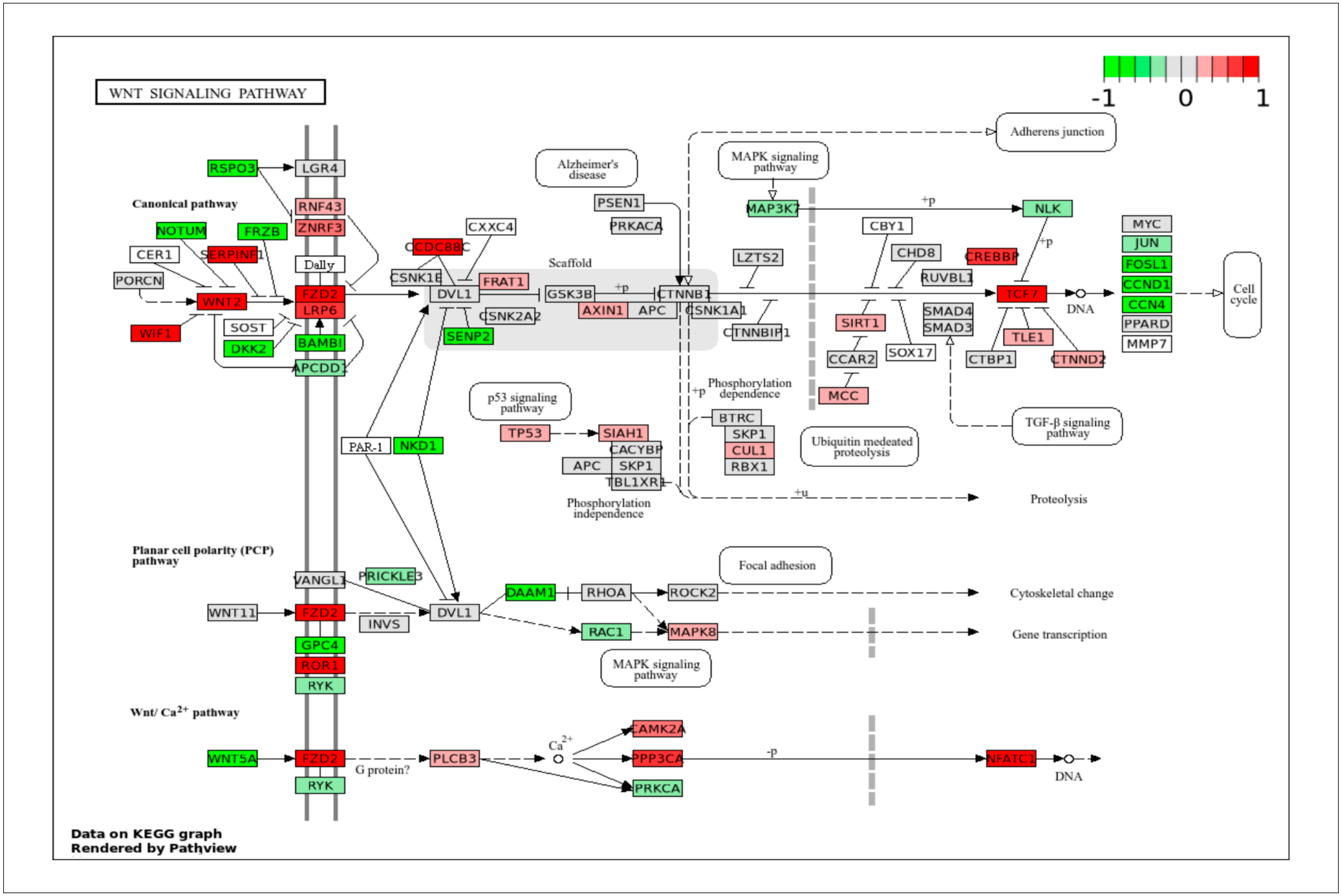

Supplemental Figure S3: KEGG Pathway of the WNT signal pathway with visualization of gene expression differences between ApSMCs and VpSMCs. Red represents high expression in ApSMCs while green represents high expression in VpSMCs.

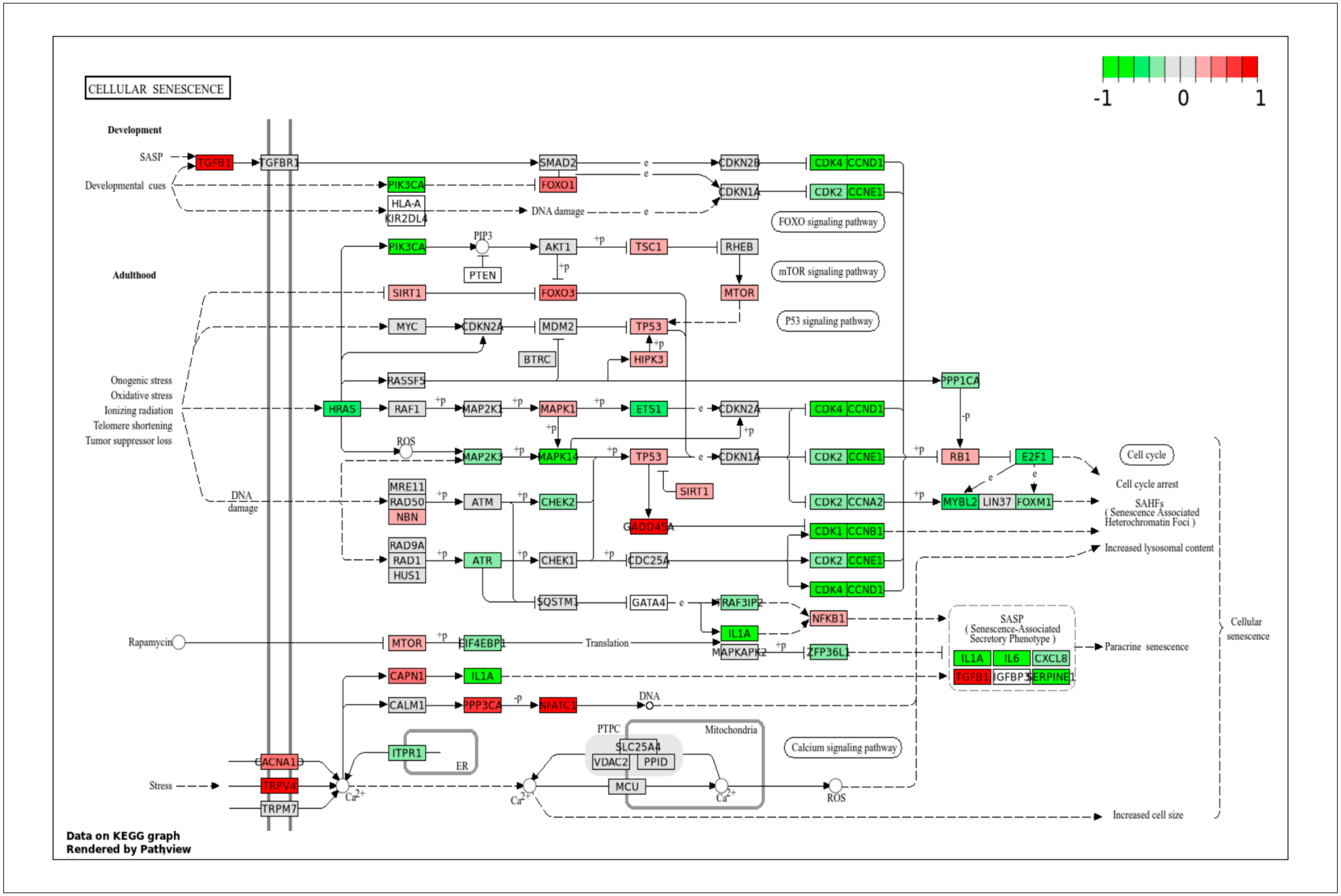

Supplemental Figure S4: KEGG Pathway of the Cellular Senescence with visualization of gene expression differences between ApSMCs and VpSMCs. Red represents high expression in ApSMCs while green represents high expression in VpSMCs.
